# A Simple, Low-cost, and Robust System to Measure the Volume of Hydrogen Evolved by Chemical Reactions with Aqueous Solutions

**DOI:** 10.3791/54383

**Published:** 2016-08-17

**Authors:** Paul Brack, Sandie Dann, K. G. Upul Wijayantha, Paul Adcock, Simon Foster

**Affiliations:** ^1^Department of Chemistry, Loughborough University; ^2^Intelligent Energy Ltd

**Keywords:** Chemistry, Issue 114, Hydrogen measurement, chemical hydrogen storage, silicon, hydrogen generation, hydrolysis, energy

## Abstract

There is a growing research interest in the development of portable systems which can deliver hydrogen on-demand to proton exchange membrane (PEM) hydrogen fuel cells. Researchers seeking to develop such systems require a method of measuring the generated hydrogen. Herein, we describe a simple, low-cost, and robust method to measure the hydrogen generated from the reaction of solids with aqueous solutions. The reactions are conducted in a conventional one-necked round-bottomed flask placed in a temperature controlled water bath. The hydrogen generated from the reaction in the flask is channeled through tubing into a water-filled inverted measuring cylinder. The water displaced from the measuring cylinder by the incoming gas is diverted into a beaker on a balance. The balance is connected to a computer, and the change in the mass reading of the balance over time is recorded using data collection and spreadsheet software programs. The data can then be approximately corrected for water vapor using the method described herein, and parameters such as the total hydrogen yield, the hydrogen generation rate, and the induction period can also be deduced. The size of the measuring cylinder and the resolution of the balance can be changed to adapt the setup to different hydrogen volumes and flow rates.

**Figure:**
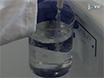


## Introduction

Due to their high energy density, lithium-ion batteries are currently one of the most popular power sources for portable consumer electronics. However, the amount of energy that can be delivered by a battery is limited. There is thus currently much interest in developing alternative methods of providing portable power. One of the more promising methods is the use of proton exchange membrane (PEM) fuel cells, which generate electricity and water by combining hydrogen and oxygen. PEM fuel cells have two main advantages over batteries. Firstly, PEM fuel cells can provide power for a much longer period of time (as long as a flow of hydrogen is maintained). Secondly, depending on the fuel source, PEM fuel cells can have a much greater energy density than batteries, meaning that a smaller system can provide more energy.^1,2^ As a result of this, there is a currently a large amount of research directed at developing portable, on-demand hydrogen sources.^2-7^ One method which is currently receiving much attention is the generation of hydrogen by reacting chemicals with water.^8,9^

One of the most important parameters which must be measured in these reactions is the evolution of hydrogen. For simple reactions, such as the evolution of hydrogen by the addition of chemical hydrogen storage materials to aqueous solutions, it is advantageous to have a simple, low cost measurement system. An example of such a system is the water displacement method, in which the volume of gas generated in a chemical reaction is measured simply by tracking the volume of water displaced from an inverted water-filled measuring cylinder. This technique originated in the pneumatic trough, which was developed by the botanist Stephen Hales and then adapted and put to its most famous use by Joseph Priestley to isolate several gases, including oxygen, in the 18^th^ century.^10,11^ The water displacement method is applicable to any gas which is not particularly soluble in water, including hydrogen, and is still widely used to record the volume of hydrogen generated from the reactions of various chemicals, such as sodium borohydride, aluminum, and ferrosilicon, with water.^12-20^

However, the classic water displacement method, involving manual recording of the changes in the water level as gas is evolved, is tedious and can, at higher gas flow rates when the water level changes rapidly, be inaccurate, as it is difficult for the experimenter to take an accurate reading. Manually recorded data is also inherently low in temporal resolution, as an experimenter cannot realistically take readings at smaller intervals than ~ 10 sec.

Several researchers have overcome this problem by using cameras to record the water displacement process and data analysis software to extract the change in volume over time.^21-25^ However, this requires knowledge of computer programming and relatively expensive equipment. Other researchers have made use of mass-flow meters to record the hydrogen flow.^26-29^ However, these are often only capable of detecting gas over a narrow range, and are better suited to applications in which the flow is maintained at a relatively constant level.

A simpler approach to obtaining higher resolution, more accurate data is to channel the water displaced by hydrogen evolution into a receiver vessel which is placed on a mass balance.^30-35^ The variation of this method described herein makes use of general laboratory grade glassware and a low-cost, commercially available balance to record hydrogen evolution from the reaction of silicon with aqueous sodium hydroxide solutions. Rather than being manually recorded, the data is logged in a spreadsheet using a data collection software package which allows the balance to send data to the computer. It should be noted that while this technique is appropriate for measuring hydrogen evolution on the milliliter scale, it is not suitable for measuring very small (due to the uncertainty in the balance) or very large (due to the limited size of the measuring cylinder) volumes of hydrogen without appropriate adaptation (*i.e.,* using a higher resolution balance or a larger measuring cylinder).

## Protocol

### 1. Set-up of Data-logging Software

1. Install the data collection and spreadsheet software onto a computer equipped with an RS232 serial port.
2. Connect the computer to the balance using an appropriate RS232 connector cable (in this method both the computer and the balance required a 9-pin connector). The balance will typically be connected to COM1.
3. Open the data collection software.
4. To log the data in a spreadsheet (*e.g.*, Excel), go to 'Mode', then 'Send keystrokes to', enter the appropriate name of the spreadsheet software in the 'Application Title Bar Text' and select 'excel.exe' in the 'Command line', then press 'OK'. A check mark should appear next to 'Send Keystrokes To' in the 'Mode' drop-down menu.
5. Go to 'Port', then 'Settings', and ensure that the values are appropriate to the balance in question, then press 'OK'.
6. Go to 'Define', then 'Define input data record structure', and select 'Numeric char received' in the 'Start Of Record Event' section and 'Carriage Return or CrLf Received' in the 'End Of Record Event' section, then press 'Continue'.
7. When a box entitled 'Input Record Structure' appears, select 'Each data record contains a single data field' and then press 'Continue'.
8. When a box entitled 'Input Record Definition Editor - Send Keystrokes Mode' appears: in Field 1, set the 'Input Filter' to 'Numeric Data Only' and the 'Field Postamble Keystrokes' to '{Tab}{Minute}:{Second}{LEFT}{DOWN}' and then press 'OK'.
9. Go to 'Define', then 'Define hot keys and hot actions'. Select Hot Key 1, then select the Hot Key Action 'Suspend WinWedge' and assign this the Hot Key Keystroke of 'BACKSPACE', and press OK.
10. Go to 'File', then 'Save As', and save the method in an appropriate folder.

### 2. Experimental Set-up

1. Add water to a glass bowl until it is approximately ¾ full. Then, place the glass bowl on a temperature-controlled stirrer-hotplate and heat to 50 °C; alternatively, use a thermostatically controlled water bath.
2. Add deionized water (5 ml) to a 50 ml round-bottomed flask and position this in the water bath such that the level of the water in the bath is well above the level of water in the flask.
3. Insert a thermometer into the neck of the round-bottomed flask to monitor the water temperature (after equilibration, the temperature of the water in the flask is usually ~ 5 °C lower than the set point on the hotplate). Note: The set-up is ready when the temperature of the water in the flask remains constant over a 10 min period.
4. Fill a beaker with deionized water.
5. Place an empty beaker onto the data-logging balance.
6. Construct a bridge from plastic sheet which can transfer water from the spout of the beaker to the empty beaker on the data-logging balance. Ensure that the plastic bridge is not in physical contact with the beaker on the data-logging balance.
7. Fill a 500 ml measuring cylinder with deionized water.
8. While covering the open end with a gloved hand, invert the measuring cylinder and place it into the beaker such that the open end of the measuring cylinder is just below the surface of the water.
9. Use a retort stand fitted with two bosses and clamps to support the measuring cylinder. Depending on the size of the measuring cylinder, place counterweights on the base of the retort stand to prevent it from falling due to the weight of the water.
10. Adjust the position of the beaker such that the spout is in contact with the plastic bridge.
11. Carefully raise the measuring cylinder to allow release of water and ingress of air to ensure that the level of air in the measuring cylinder is consistent at the beginning of each experiment (for example, 100 ml of air).
12. Insert the non-ground glass joint end of a modified adapter into a length of tubing. Seal by carefully wrapping Parafilm around the connection between the joint and the tubing.
13. Insert the end of the tubing into the measuring cylinder.
14. Ensure that the addition of excess water will result in it running off onto the balance by adding some water to the beaker. Leaks can occur at high flow rates at the connection between the beaker's spout and the plastic bridge.
15. Ensure that the balance does not read zero. If necessary, add some water to the beaker on the data-logging balance.
16. Using a balance, weigh out either 0.05, 0.10, 0.15, 0.20, or 0.25 g of silicon into a small glass vial; do not use a plastic weighing boat as some silicon tends to be trapped on the inner neck of the flask when it is added to the reaction mixture from a weighing boat. This problem is avoided by instead rapidly inverting a small glass vial into the neck of the flask.

### 3. Experimental Procedure

1. Add sodium hydroxide solution (5 ml, 20 wt%) to a 50 ml round-bottomed flask and position this in the water bath such that the level of the water in the bath is well above the level of water in the flask.
2. Insert a thermometer into the neck of the round-bottomed flask to monitor the solution temperature (after equilibration, the temperature of the water in the flask in this set-up is usually ~ 5 °C lower than the set point on the hotplate).
3. Leave for 10 min to equilibrate.
4. Before the equilibration period ends, open a new spreadsheet in the spreadsheet software package and then open the data collection software. Load the method created in Step 1 by going to 'File' on the data collection software start menu, and then 'Open method'.
5. Just before the 10 min equilibration period is due to end, go to 'Activate' and then click on 'Normal mode'. Data will start being logged in the spreadsheet software package.
6. At the end of the 10 min equilibration period, add the silicon by rapidly inverting the glass vial and depositing the silicon into the sodium hydroxide solution.
7. Rapidly place the ground glass joint of the adapter which is attached to the tubing into the neck of the round bottomed flask. Zero the balance. The moment at which the balance is zeroed will be taken as time (t) = 0 in the data analysis.
8. After 10 min have elapsed, stop the data logging by pressing the backspace key and then selecting the 'Quit' option on the data collection software menu. Save the file in the spreadsheet software package.
9. Remove the adapter which is attached to the tubing from the round-bottomed flask and add water to quench the reaction.
10. Isolate the solid residue in the flask for further analysis by centrifugation or gravity filtration, or transfer the entire reaction mixture to a beaker and neutralize with hydrochloric acid (1 M) and dispose of the waste appropriately.

### 4. Data Analysis

1. Ensure that the data is loaded into an appropriate spreadsheet software package.
2. Find the point at which the balance is zeroed; this is considered to be the (t) = 0 point of the reaction.
3. Delete the data which precedes this.
4. Insert a column to the left of this data. This will contain the time.
5. Add appropriate time intervals, starting from zero, to the column which has just been inserted. The balance used in these studies logged 8.5 data points per second, and thus time intervals of 0.117647 (=1/8.5) sec were used.
6. Consider gas which has been collected over water to be saturated with water vapor. During the collection process, the water level in the measuring cylinder adjusts to maintain the internal pressure in the measuring cylinder at atmospheric pressure.
7. Apply an approximate correction factor using Dalton's Law, which states that the sum of the individual partial pressures of the gases in a mixture (P_1_…P_n_) is equal to the total pressure (P_tot_). As, if the room temperature is 298 K, the partial pressure of water vapor is 31,69.9 Pa, and the total pressure of gas in the measuring cylinder is atmospheric pressure (101,325 Pa), it can be calculated that there is approximately 3.08% water vapor by volume in the collected gas. Estimate the amount of water vapor in the hydrogen at other temperatures by using the partial pressure of water vapor at the temperature in question.
8. To obtain an estimate of the amount of hydrogen generated (if the room temperature is 298 K), multiply the gas volume by 0.97.
9. Estimate the initial hydrogen generation rate by fitting a linear trend line to the initial steep slope of the hydrogen generation curve.
10. Take the induction period as the time taken for water to be displaced from the measuring cylinder. These estimates of induction period are not absolute; the actual hydrogen generation reaction starts before the end of the 'induction period' estimated in these experiments as a certain amount of hydrogen must be generated to be able to begin displacing water. However, these values do allow for an assessment of the relative change in induction period between experiments.

## Representative Results

To investigate the reproducibility of the experimental set-up, varying masses of silicon were reacted with aqueous sodium hydroxide solutions to generate hydrogen. Each reaction was performed in triplicate. The average hydrogen generation curves are shown in **Figure 1**. Average total hydrogen yields, hydrogen generation rates, and induction periods for each mass of silicon were also calculated and are plotted with error bars representing one standard deviation in **Figures 2, 3**, **and 4**, respectively. There was very little deviation in the total hydrogen yields and hydrogen generation rates between reactions, and a greater level of deviation in the induction periods.


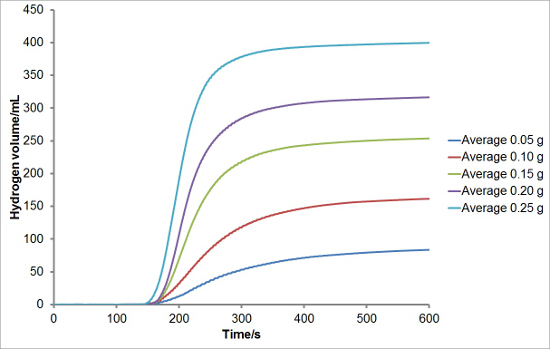
**Figure 1:Example of Hydrogen Generation Curves from the Reaction of Silicon with Aqueous Sodium Hydroxide.** Various masses of silicon (0.05, 0.10, 0.15, 0.20 and 0.25 g) were reacted with aqueous sodium hydroxide solution (5 ml, 20 wt%) at 50 °C. Hydrogen generation was recorded for a period of 10 min. The reactions were conducted in triplicate and the results averaged. Please click here to view a larger version of this figure.


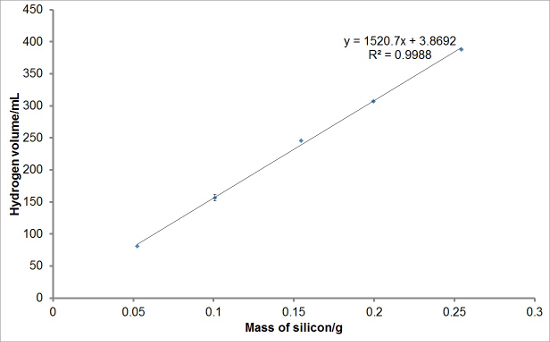
**Figure 2:Example of Hydrogen Yield Values from the Reaction of Silicon with Aqueous Sodium Hydroxide.** The total volumes of hydrogen evolved in 10 min were deduced from the hydrogen generation curves. The average total hydrogen yields for each mass of silicon were obtained and plotted. It can be seen that there is a linear relationship between the mass of silicon used in the reaction and the volume of hydrogen generated under these reaction conditions. The error bars represent one standard deviation of the total hydrogen yields. Please click here to view a larger version of this figure.


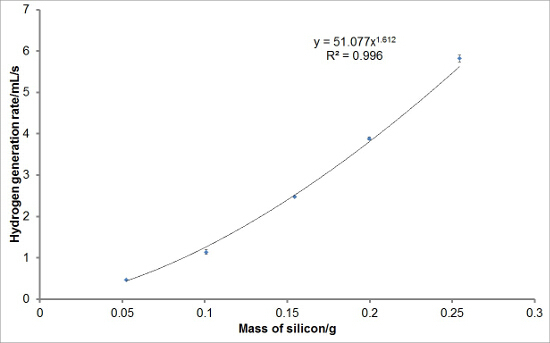
**Figure 3:Example of Hydrogen Generation Rate Values from the Reaction of Silicon with Aqueous Sodium Hydroxide.** The initial or maximum rates of hydrogen generation for each mass of silicon were calculated from the hydrogen generation curves. The average initial or maximum hydrogen generation rates for each mass of silicon were obtained and plotted. It can be seen that, on average, there is a power relationship between the mass of silicon used in the reaction and the initial or maximum hydrogen generation rate observed under these reaction conditions. The error bars represent one standard deviation of the initial or maximum hydrogen generation rates. Please click here to view a larger version of this figure.


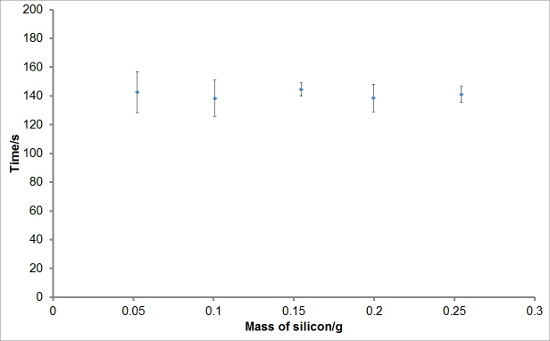
**Figure 4:Example of Induction Period Values from the Reaction of Silicon with Aqueous Sodium Hydroxide.** The induction periods for the hydrogen generation for each mass of silicon were deduced from the hydrogen generation curves. The average induction period for each mass of silicon was obtained and plotted. It can be seen that, on average, there is no great change in the induction period between the experiments. The error bars represent one standard deviation of the initial or maximum hydrogen generation rates. Please click here to view a larger version of this figure.

**Figure 5** shows some representative results from a sub-optimal experiment. In this case, the low flow of hydrogen between 200 and 800 sec results in the build-up of drips due to the surface tension of the water, which fell at approximately 400 and 710 sec. Though these drips do not affect the calculation of the maximum hydrogen generation rate, they could have an effect on the total hydrogen yield if, for example, the measurement was stopped before the drip fell. It is thus necessary to either alter the reaction conditions (in this case, for example, by adding a greater mass of aluminum-silicon alloy or using a higher concentration of sodium hydroxide) to ensure a higher flow of gas or the reaction setup to prevent the buildup of drips.


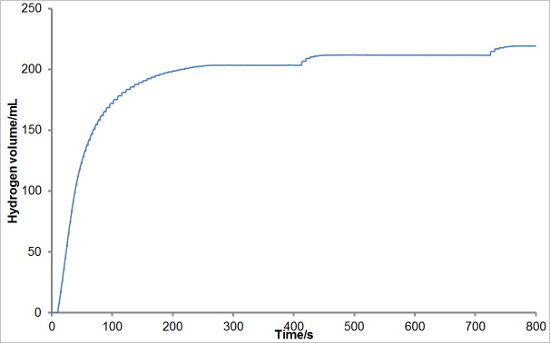
**Figure 5: Example of a Sub-optimal Experiment.** In this experiment, aluminum (65.7%)-silicon (34.3%) alloy (0.2 g) was reacted with aqueous sodium hydroxide solution (5 ml, 10 wt%) at 40 °C. Though at the initial high rates of hydrogen evolution the recording of the hydrogen generation is optimal, as the flow slows the surface tension of the water results in drips being formed. The drips fall at approximately 400 and 710 sec, in this case. Please click here to view a larger version of this figure.

## Discussion

The most critical steps of the protocol are those which occur at the beginning of an experiment. The large temperature dependence of the rate of these hydrolysis reactions means that great care must be taken to ensure that the solution temperature has reached equilibrium before the addition of the solid. The solid must be added rapidly and completely, the ground glass joint of the adapter must be properly inserted into the neck of the round-bottomed flask, and the balance must then be zeroed as rapidly as possible. An incorrect measurement of start time and reaction temperature will generate incorrect results.

The method does have some limitations. It is imperative that the beaker into which the measuring cylinder is inserted is as narrow as practicable to ensure that the water displaced from the measuring cylinder is rapidly channeled down the plastic bridge onto the balance. Otherwise, the surface tension of the water allows for a slow buildup of the water level at low flow rates (see **Figure 5**) until the point at which all of the water is released in a large drip.

The error of the balance also limits the resolution of the data. In these experiments, a balance with an error of ± 0.05 g was used, which is adequate when generating several hundred milliliters of hydrogen, but a balance with a smaller error would be required if smaller volumes were being measured.

As the displaced water drips from the bridge onto the balance, the mass recorded by the balance oscillates,* i.e.*, as a drip falls onto the balance, the balance momentarily records a slightly larger mass. This means that differentiation of high time resolution raw data using software packages is problematic as the gradient oscillates. The most appropriate way to find the gradient of the steepest part of the hydrogen generation curve, and thus the hydrogen generation rate, is to fit a straight line to it and calculate its gradient.

By automatically logging the data in a spreadsheet, this method offers a significant improvement in accuracy and temporal resolution with respect to water displacement methods which rely on recording the volume of gas evolved manually. However, though it is considerably lower in cost than methods which use cameras and image analysis software to track gas evolution, it is generally lower in temporal resolution, and such camera-based methods also avoid the problem of oscillating mass-balance readings due to water forming drops and therefore produce data which can be more easily processed by differentiation.

The water displacement method is applicable to the collection of any gas that has low solubility in water. Thus, this experimental protocol could be modified for the measurement of rates of gas generation from other chemical reactions which evolve poorly water soluble gases.

## Disclosures

The authors have nothing to disclose.
